# The Empty-Nest Power User Management Based on Data Mining Technology

**DOI:** 10.3390/s23052485

**Published:** 2023-02-23

**Authors:** Jing Li, Jiahui Yang, Hui Cai, Chi Jiang, Qun Jiang, Yue Xie, Zimeng Lu, Lingzhi Li, Guanqun Sun

**Affiliations:** 1 College of Mechanical and Electrical Engineering, China Jiliang University, Hangzhou 310018, China; 2 Electric Power Research Institute, State Grid Zhejiang Electric Power Co., Ltd., Hangzhou 310007, China; 3 College of Modern Science and Technology, China Jiliang University, Yiwu 322002, China

**Keywords:** abnormal power consumption detection, analysis of power consumption behavior, empty-nest user identification, data mining

## Abstract

With the aging of the social population structure, the number of empty-nesters is also increasing. Therefore, it is necessary to manage empty-nesters with data mining technology. This paper proposed an empty-nest power user identification and power consumption management method based on data mining. Firstly, an empty-nest user identification algorithm based on weighted random forest was proposed. Compared with similar algorithms, the results indicate that the performance of the algorithm is the best, and the identification accuracy of empty-nest users is 74.2%. Then a method for analyzing the electricity consumption behavior of empty-nest users based on fusion clustering index adaptive cosine K-means was proposed, which can adaptively select the optimal number of clusters. Compared with similar algorithms, the algorithm has the shortest running time, the smallest Sum of the Squared Error (SSE), and the largest mean distance between clusters (MDC), which are 3.4281 s, 31.6591 and 13.9513, respectively. Finally, an anomaly detection model with an Auto-regressive Integrated Moving Average (ARIMA) algorithm and an isolated forest algorithm was established. The case analysis shows that the recognition accuracy of abnormal electricity consumption for empty-nest users was 86%. The results indicate that the model can effectively detect the abnormal behavior of empty-nest power users and help the power department to better serve empty-nest users.

## 1. Introduction

Aging is one of the main trends of the current world population structure. With the growth of life expectancy, the decline of population fertility, and the deepening of the degree of aging, the number of empty-nesters is constantly increasing [1]. As the largest developing country, China’s population aging is characterized by its rapid speed and large scale [2]. According to the National Bureau of Statistics, the number of empty-nesters reached 133 million in 2021 and is expected to exceed 200 million by 2035 [3,4]. In the current situation of such a large population, the current government and society lack effective technical means to identify the empty-nest elderly, which brings great challenges to the government’s accurate support for empty-nest users. Empty-nesters often forget to turn off electrical appliances and misuse electrical appliances due to memory loss and mobility problems, causing serious consequences. Therefore, it is necessary to use big data mining technology to carry out research on empty-nest power user identification and power consumption behavior and realize the fine classification of empty-nest power users. At the same time, the research results can be used as the decision-making basis for power companies to provide customers with more refined services.

At present, the analysis of user classification and power consumption behavior is basically focused on the division between industries, and rarely accurate to a certain kind of users. Although some scholars have conducted research on empty-nest elderly identification by using mobile phone communication data [5,6,7], the communication data used in this method may easily lead to user privacy issues. Most electricity behavior research focused on using the big data of the power user side. Based on the selected features and weights, the clustering methods are utilized to conduct a similarity search on samples. The common way is to calculate the similarity according to the load curve or load characteristic index then using regression method, clustering algorithm and fuzzy algorithm for classification. The classification methods include the partition-based clustering methods such as K-means [8,9], and K-Medoids [10,11], the model-based methods such as COBWEB, self-organizing neural network [12,13], the density-based methods such as DBSCAN [14], and the clustering methods such as fuzzy clustering [15] and hierarchical clustering. However, these methods are prone to the problems of unstable clustering results, slow speed, and poor effect when dealing with high-dimensional and massive load curves with large differences in cluster shapes. Researchers often combine dimensionality reduction algorithms to improve them. Wang et al. [16] used the load curve clustering method based on an unsupervised extreme learning machine, Chen et al. [17] improved the sample local density and distance calculation criteria of the original clustering algorithm, Lin et al. [18] re-expressed load data with the variable temporal resolution based on information entropy piecewise aggregation approximation, Wang et al. [19] used a Markov model to model power consumption, transformed the load curve data set into multiple state transition matrices, and classified users into multiple clusters using fast search and density peaks.

Based on the above status quo, this paper proposes a management method for empty-nest users based on data mining technology, including empty-nest user identification, power consumption behavior analysis, and abnormal power consumption detection. The big data analysis method is used to mine the potential electricity consumption characteristics of the empty-nest elderly, focusing on the analysis of the differential characteristics of the electricity consumption behavior of empty-nest and non-empty-nest users. An empty-nest power user identification model based on the weighted random forest algorithm is constructed to realize the identification of empty-nest users. The typical load curve of the user is extracted using the adaptive cosine K-means clustering method based on the clustering index fusion. This method can adaptively select the optimal number of clusters through machine learning, avoiding the situation where the number of types of daily load curves of power users is unknown. In this case, due to the inappropriate setting of the number of clusters artificially, the deviation of a single clustering result is too large, thereby improving the accuracy of load classification. Based on the past electricity consumption data of empty-nest users, ARIMA and the isolated forest algorithm are used to establish an abnormal electricity consumption detection model to detect the daily life and electricity consumption behavior of empty-nest elderly people from the perspective of electricity consumption, which will help the power sector to better avoid the electricity consumption risk of such users.

The remainder of this paper is organized as follows. Section 2 analyzes and extracts the electricity consumption characteristics of empty-nest users, and introduces the proposed empty-nest user identification algorithm based on the weighted random forest algorithm. Section 3 proposes a research method based on clustering index fusion adaptive cosine K-means clustering of electricity consumption behavior of empty-nest users. Section 4 proposes two abnormal electricity consumption detection models for the two types of abnormal electricity consumption behaviors common to empty-nest users. Section 5 deploys the proposed model online for instance verification and result analysis. Section 6 summarizes the work of this paper and makes some suggestions for further research in the future.

## 2. Research on Empty-Nest Power User Identification Based on Data Mining

### 2.1. Based on the Analysis of Electricity Consumption Characteristics of Empty-Nest Elders

**Figure 1:**
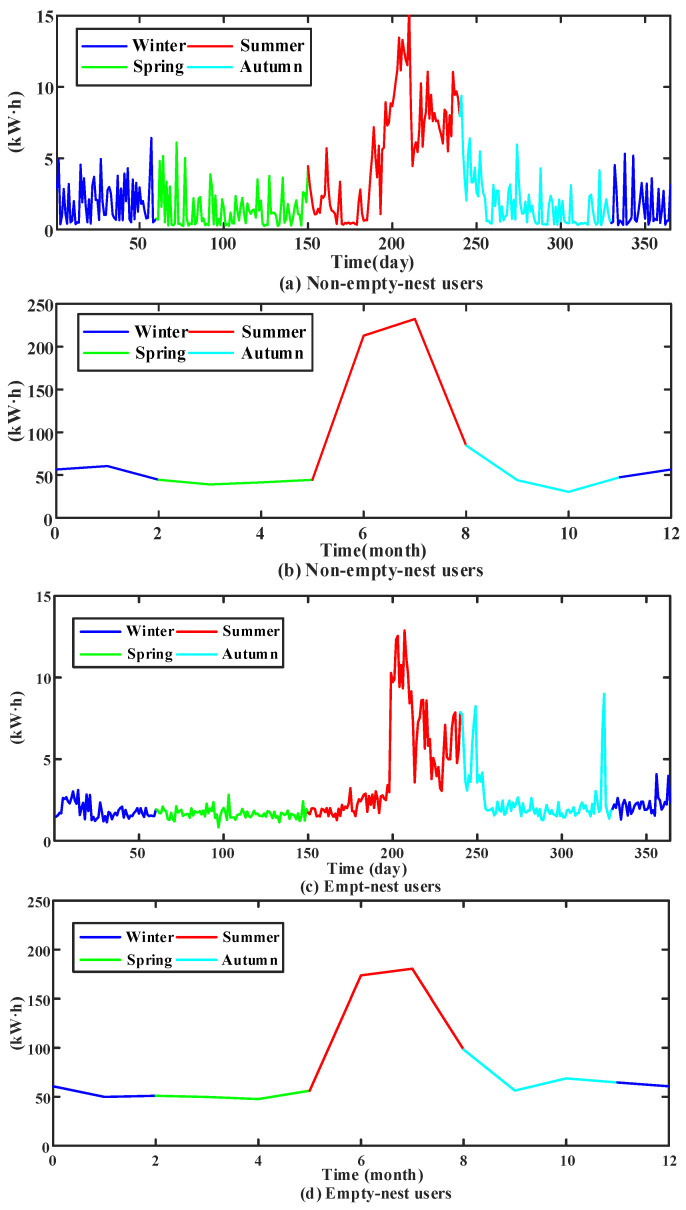
Electricity consumption pattern of empty-nest users and non-empty-nest users.

The energy consumption levels of non-empty-nest users and empty-nest users are often quite different. Their average electricity consumption level, the peak-valley value of electricity consumption, electricity fluctuation, and seasonal electricity consumption trends are different. However, factors involving household population and household income of users affect their electricity consumption level. For example, there are very few non-empty-nest users and empty-nest users whose electricity consumption laws are relatively similar, as shown in Figure 1.

**Figure 2:**
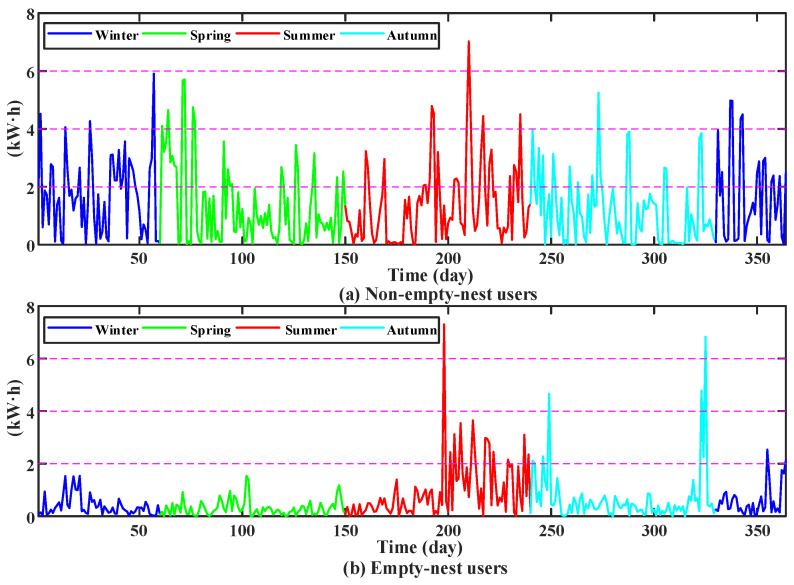
Chart of electricity consumption after differential treatment.

The daily and monthly electricity consumption of the non-empty-nest users and the empty-nest users shown in Figure 1 are relatively similar. Hence, the simple clustering method cannot effectively distinguish the two. The orderly arrangement of users’ electricity consumption and time constitutes a time series. In the time series model, the difference method is commonly used to repair the data, and the electricity consumption is processed by difference. From the perspective of the fluctuation change of users’ electricity consumption in the two adjacent days, the potential distinguishing features of similar users are explored. The power characteristics of the above users after daily power difference processing are shown in Figure 2.

As can be seen from Figure 2, the fluctuation amplitude of daily differential electricity of non-empty-nest users (Figure 2a) is large in a year. For empty-nest users (Figure 2b), the amplitude of differential electricity fluctuation is large in summer, but small for the rest of the time.

### 2.2. Feature Extraction of Electricity Consumption

Firstly, after the user power consumption difference processing, the population distribution of the user group daily power difference in different daily difference power consumption intervals is investigated. Suppose *S* is the maximum daily differential power consumption for all empty-nesters in the training set *N*_1_ in a year, and then the differential power consumption is divided according to the interval [*P_i_*, *P_i_*_+1_] (*i* = 1, 2, … *l*), where *P_i_*_+1_ = *P_i_*_+2_, the initial value *P*_1_ = 0, *P_i_* is divided into *P*_1_~*P*_2_, *P*_2_~*P*_3_, … and other intervals, calculate the distribution of users in each interval as the power consumption feature, and in terms of power consumption level, introduce the maximum annual, quarterly and monthly power consumption, The minimum value and the difference value are used to characterize the user’s power consumption level.

In terms of electricity fluctuation, the normalized electricity fluctuation dispersion *Cv* and standard deviation σ are introduced to jointly characterize the degree of user electricity fluctuation:

(1) $$\left\{ \begin{array}{l} \;u=\frac{\sum_{d=k_{1}}^{k_{2}}x_{d}}{k_{2}-k_{1}+1}\\ \sigma (d)=\sqrt{\frac{\sum_{d=k_{1}}^{k_{2}}{\left(x_{d}-u\right)}^{2}}{k_{2}-k_{1}+1}} \end{array}\right.\;(1\leq k_{1}<k_{2}\leq 365)$$

(2) $$Cv(d)=\frac{\sigma }{u}$$

where *u* is the average daily electricity consumption with the electricity time length of *k*_2_ − *k*_1_ + 1, *σ*(*d*) is the standard deviation of daily electricity consumption with the electricity time length of *k*_2_ − *k*_1_ + 1, and *Cv*(*d*) is the dispersion of daily electricity consumption with the electricity time length of *k*_2_ − *k*_1_ + 1.

In terms of the electricity consumption trend, the average electricity consumption difference *T_d_*, and the ratio *T_s_* two adjacent months are introduced to reflect the electricity consumption trend of users:

(3) $$\left\{ \begin{array}{l} T_{d}=\left|{\bar{y}}_{m}-{\bar{y}}_{m+1}\right|\\ T_{s}=\frac{{\bar{y}}_{m}}{{\bar{y}}_{m+1}} \end{array}\right.$$

### 2.3. Empty-Nest User Identification Algorithm Based on Weighted Random Forest

Since the ratio of non-empty-nest and empty-nest users is close to 10:1, there is a severe data imbalance in the ratio of the two. The degree of mixed samples in the data set is relatively low, which may lead to data sensitization [20]. The weighted random forest (WRF) algorithm is used to improve the learning and recognition ability of random forests for a few samples.

In this paper, weights are applied to the two processes of decision tree construction and final voting. In the construction of decision trees, the weighted Gini index is used to find the optimal split. In the final prediction results, the “weighted majority voting” decision is adopted, that is, the results of each tree are assigned to the weighted voting.

1. Weighted Gini Decision Tree Construction

The bootstrap self-sampling method is used to extract the data set *D_N_*_*×M*_ from the original data. The data set *D* is composed of *N* training samples and *M* features. The weight *W_k_* of each sample in the data set *D* is inversely proportional to the frequency *P_k_* (*k* = 1, 2, … *C*) of this classification in the sample set, and *C* is the number of sample categories.

(4) $$W_{k}=\frac{1}{P_{k}}\;\;\;\;(k=1,2,\ldots C)$$

The weighted Gini index *GW*(*D*) of sample dataset *D* is:

(5) $$\left\{ \begin{array}{l} GW(D)=1-\sum_{k=1}^{C}S_{k}{}^{2}\\ S_{k}=\frac{I_{k}*W_{k}}{\sum_{k=1}^{C}I_{k}*W_{k}} \end{array}\right.$$

In Equation (5), *I_k_* is the number of category *k* in sample *D*, *S_k_* is the weighted proportion of class *k* sample, and *GW*(*D*) is the Gini value of the data set *D* after weighting.

It is assumed that the data set *D* can be divided into two parts *D*_1_ and *D*_2_ according to feature a, and the minimum value of *GW*(*D*, *a*) is obtained to obtain the optimal partition to construct the weighted decision tree.

(6) $$GW(D,a)=\frac{D_{1}}{D}GW(D_{1})+\frac{D_{2}}{D}GW(D_{1})$$

The minimum value of *GW*(*D*, *a*) is the optimal feature of the node, and *a* (*a* ∈ *M*) is the splitting feature.

2. Decision Based on Weighted Majority Voting

Since the random forests have the extracted data back in the self-service sampling method, the probability that each sample fails to be extracted is:

(7) $$P={\left(1-\frac{1}{N}\right)}^{N}$$

When *N* → ∞, *P* is about 0.37, so nearly 37% of the data in each decision tree is not extracted. This data is called out-of-back (OBB) data, then the weight of a single decision tree *t* (*t* = 1, 2, … *T*) is the prediction accuracy *acc_t_* of the current decision tree model on the OOB dataset, and the final prediction result of the random forest is:

(8) $$\left\{ \begin{array}{l} t_{k}=\frac{W_{k}*leaf(k)}{\sum_{k=1}^{C}W_{k}*leaf(k)}\\ f_{WRF}(k)=\frac{\sum_{t=1}^{T}acc_{t}*t_{k}}{\sum_{t=1}^{T}acc_{t}} \end{array}\right.$$

In Equation (8), *leaf*(*k*) is the number of cases belonging to category *k* on the output node of the decision tree *t*-*leaf*, *t_k_* is the probability that the decision tree *t* is predicted to be category *k*, and *f_WRF_*(*k*) is the probability that the weighted vote is predicted to be category *k*.

While predicting users in the test set, if the probability *f_WRF_* (empty-nest) of the test sample identification result is greater than the threshold *α* ∈ (0, 1), it is determined that the user is an empty-nest user, otherwise, it is a non-empty-nest user.

(9) $$\left\{ \begin{array}{l} f_{WRF}(\mathrm{empty}\;\mathrm{nest})>\alpha \;\;\;\;\mathrm{Determined}\;\mathrm{as}\;\mathrm{empty}\;\mathrm{nest}\;\mathrm{user}\\ f_{WRF}(\mathrm{empty}\;\mathrm{nest})\leq \alpha \;\;\;\;\mathrm{Determined}\;\mathrm{as}\;\mathrm{non}-\mathrm{empty}\;\mathrm{nest}\;\mathrm{user} \end{array}\right.$$

## 3. Research on Electricity Consumption Behavior of Empty-Nest Users Based on Clustering Index Fusion Adaptive Cosine K-Means Clustering Method

### 3.1. Cosine K-Means Algorithm

K-means is an iterative clustering algorithm. Common K-means algorithms regard Euclidean distance as a similarity evaluation index. The Euclidean distance focuses on the numerical difference in data, which cannot reflect the similarity between data changes well. In the study of the electricity consumption mode of the empty-nest elders, the electricity consumption mode is easily affected by seasonal factors. Hence, it may be affected by seasonal electricity factors when using the Euclidean distance evaluation index. This paper adopts the cosine similarity evaluation index [21]. Compared with the Euclidean distance, the cosine similarity index focuses more on the similarity of the change rule of the clustering vector, which can reduce the impact of seasonal electricity consumption by users. The calculation equation for cosine similarity [22] is as follows:

(10) $$\cos(A,B)=\frac{A\cdot B}{\left\|A\right\|\left\|B\right\|}=\frac{\sum_{i=1}^{n}x_{i}y_{i}}{\sqrt{\sum_{i=1}^{n}x_{i}{}^{2}}\times \sqrt{\sum_{i=1}^{n}y_{i}{}^{2}}}$$

In the Equation (10), *A* = {*x_i_*|*i* = 1, 2, … *n*} and *B* = {*y_i_*|*i* = 1, 2, … *n*} are two power curve vectors.

Cosine similarity pays more attention to the classification of curves with similar power consumption rules, so using it as an evaluation index can reduce the impact of seasonal power consumption by users. Assume a user’s electricity consumption data set *D* = {*d*_1_, *d*_2_, … *d_n_*} ∈ *R^s^*, *D* is an *n*-dimensional *s* metadata set, *n* is the number of days the user has used electricity in the past, and *s* is the load current data of the user at 96 points per day, the cosine K-means clustering algorithm steps are as follows:

1. For the initialization of the cluster center, to avoid K-means falling into the local optimum due to improper selection of the initialization center, this paper randomly selects *k* samples *d*_1_, *d*_2_, … *d*_k_ ∈ *R^s^* with a long cosine similarity distance as the initial cluster center
2. Calculate the cosine similarity between the remaining samples and the initial cluster center, classify it into the cluster with the closest similarity, and use the average vector of each cluster as the new cluster center *C_j_* (*j* = 1, 2, … *k*).
  (11) $$C_{j}=\frac{1}{n_{j}}\sum_{d_{i}}d_{i}$$

In the Equation (11), *n_j_* is the number of samples in cluster *j*, and *d_i_* is the sample belonging to class *C_j_*.

3. Repeat step (2) and iterate continuously until the criterion function, i.e., SSE converges. The calculation equation [23] is:
  (12) $$\mathrm{SSE}=\sum_{j=1}^{k}\sum_{d_{i}\in C_{j}}{\left|\cos\left(d_{i},C_{j}\right)\right|}^{2}$$

**Figure 3:**
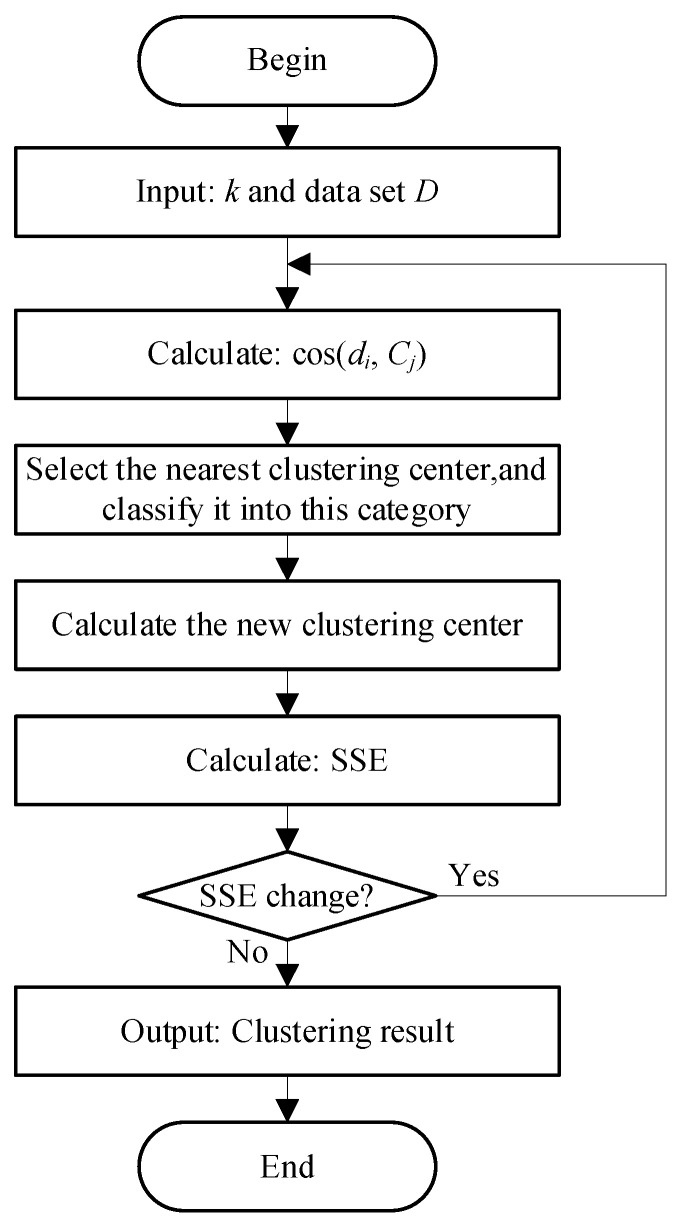
Flow chart of cosine K-means algorithm.

The flow chart of the cosine K-means algorithm is shown in Figure 3.

### 3.2. Clustering Algorithm Evaluation Index

1. SC Evaluation Index

The SC index, also known as the average silhouette coefficient, reflects the degree of cohesion of the clustering results and the dispersion between various types and uses the degree of cohesion and separation in the cluster to evaluate the reasonable degree of clustering.

(13) $$\left\{ \begin{array}{l} q_{i}=(b_{i}-a_{i})/\max(a_{i},b_{i})\\ \mathrm{SC}(i)=\frac{1}{N}\sum_{1}^{N}q_{i} \end{array}\right.$$

where *a_i_* is the clustering cohesion, indicating the average distance from sample *i* to other samples in the same cluster. *b_i_* is the clustering separation degree, which represents the minimum average distance from sample *i* to other samples in different clusters. *q_i_* is the contour coefficient of the vector *i*. *N* is the number of samples. SC is the average contour coefficient. The larger the average contour coefficient SC is, the better the clustering effect is.

2. DBI Evaluation Index

The DBI index, also known as the classification accuracy index, uses the ratio of the average distance within the cluster to the distance between the clusters as the evaluation index of clustering effectiveness. The smaller the DBI value of the clustering result is, the better the clustering effect is.

(14) $$\mathrm{DBI}=\frac{1}{k}\sum_{i,j=1}^{k}\underset{i\neq j}{\max}\frac{avg(db_{i})+avg(db_{j})}{DB_{ij}}$$

In Equation (14), *k* is the number of clusters, *avg*(*db_i_*) is the average distance from all samples in cluster *i* to the cluster center, and *DB_ij_* is the distance from the cluster center of cluster *i* and cluster *j*.

### 3.3. Adaptive Cosine K-Means Algorithm Based on TOPSIS Algorithm and Clustering Evaluation Index

The TOPSIS algorithm is a multi-attribute comprehensive decision-making method [24]. In this paper, the TOPSIS algorithm is used to integrate the two indicators of SC and DBI, to realize the comprehensive evaluation of the clustering effect.

Because empty-nest users have characteristics including complexity, variability, and uncertainty, different empty-nest users have different typical electricity behavior habits. This results in different initial clustering numbers *k* of different users. Therefore, it is necessary to analyze the clustering validity under different initial clustering numbers *k* to obtain the optimal clustering number.

In the search for the optimal number of clusters, the range of the initial cluster *k* value is first determined, and the initial cluster number *k* = 2 is used for clustering. The sample number *P_i_* in each class is calculated. If *P_i_* is not less than the threshold *α*, the cluster number *k* = *k* + 1 is re-clustered until any *P_i_* is less than the threshold *α*, and the clustering is stopped. The range of the cluster *k* value is set to be 2~*N*.

For different clustering numbers *k* = 1, 2, … *N*, the average contour coefficient SC and DBI indexes are calculated, respectively. The DBI index is processed positively according to Equation (15) to construct the evaluation index matrix **X**.

(15) DBI=max(DBI)−DBI

(16) $$X=\left[\begin{array}{cc} x_{11} & x_{12}\\ \vdots & \vdots \\ x_{i}{}_{1} & x_{i2}\\ \vdots & \vdots \\ x_{N1} & x_{N2} \end{array}\right]=\left[\begin{array}{cc} {\mathrm{SC}}_{1} & {\mathrm{DBI}}_{1}\\ \vdots & \vdots \\ {\mathrm{SC}}_{i} & {\mathrm{DBI}}_{i}\\ \vdots & \vdots \\ {\mathrm{SC}}_{N} & {\mathrm{DBI}}_{N} \end{array}\right]\;\;(i\in (1,2,\cdots ,N))$$

Standardize the evaluation index matrix **X** to obtain the standardized *z_ij_* and find the positive ideal optimal solution *Z*^+^ and the negative ideal optimal solution *Z*^−^ for each column.

(17) $$\left\{ \begin{array}{l} z_{ij}=x_{ij}/\sqrt{\sum_{i=1}^{N}x_{ij}^{2}}\\ Z^{+}=(Z_{1}^{+},Z_{2}^{+})=(\max\left\{z_{1j},z_{2j},\cdots z_{Nj}\right\})(j=1,2)\\ Z^{-}=(Z_{1}^{-},Z_{2}^{-})=(\min\left\{z_{1j},z_{2j},\cdots z_{Nj}\right\}) \end{array}\right.$$

According to the positive and negative optimal ideal solutions, calculate the comprehensive evaluation score *f_i_* when the number of clusters is *k* = *i* (*i* = 1, 2, … *N*), 0 ≤ *f_i_* ≤ 1, the larger the *f_i_*, the better the clustering effect. The value of *i* when *f_i_* is the largest is selected as the optimal number of clusters *k*.

(18) $$f_{i}=\frac{\sqrt{\sum_{j=1}^{2}{\left(Z_{j}^{-}-z_{ij}\right)}^{2}}}{\sqrt{\sum_{j=1}^{2}{\left(Z_{j}^{+}-z_{ij}\right)}^{2}}+\sqrt{\sum_{j=1}^{2}{\left(Z_{j}^{-}-z_{ij}\right)}^{2}}}(i\in (1,2,\cdots ,N))$$

## 4. Research on Monitoring Abnormal Electricity Consumption of Empty-Nest Users

### 4.1. Abnormal Power Consumption Types of Empty-Nest Users

**Figure 4:**
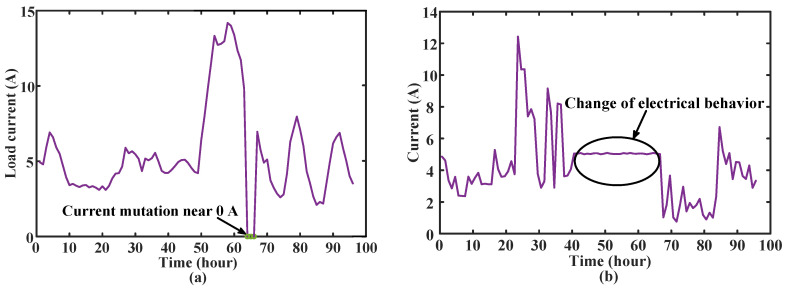
The two abnormal electricity consumption types of empty-nest users, i.e., (**a**) Current mutation near 0 A and (**b**) Change of electrical behavior.

According to the definition of the power company, there are two types of abnormal power consumption for empty-nest users. The first type is that the sudden change in the user’s load current draws near 0 A; the second type is the abnormal user’s electrical behavior. The research in this section aims to accurately detect these two types of abnormal power consumption behaviors for further troubleshooting by the staff of the power company. Examples of the two types of abnormal power usage are shown in Figure 4.

For the first type of power consumption anomaly, the analysis in Figure 4a shows that the load current of the empty-nest user suddenly dropped to 0 A at the time point 64–68, and the abnormal label given by the power company was that the rice cooker was short-circuited and the power was cut off. During the period, the current suddenly changes to 0 A. For the second type of power consumption anomalies, the analysis in Figure 4b shows that the load current of the user is close to stable at time points 41–67. The abnormal label given by the power company is forgetting to turn off high-power appliances such as electric kettles for a long time. Only electrical appliances such as electric kettles work, and the current is nearly stable during this period.

### 4.2. Zero-Crossing Detection Model of Load Current Based on ARIMA Algorithm

The user load current data collection occurs every 15 minutes and a total of 96 points are collected every day. If the load current data of the empty-nest users is tracked and forecasted in real time, each user would need to call the ARIMA model 96 times a day. Moreover, the ARIMA data of the model should be updated each time [25]. In addition, the empty-nest user base is relatively large. It is unreasonable to adopt the method of real-time tracking and forecasting every day. Therefore, this article uses the following methods to solve the problem:

1. The Opening Condition of User Load Current Zero-Crossing Anomaly Detection

Assume that the lowest load of the empty-nest user is 25 W incandescent lamp. When the user’s load current at time *N* is lower than 0.12 A, the load current zero-crossing abnormality detection will be turned on for the user. Retrieve the load data of the user in the past week, establish an ARIMA model to predict the load current at time *N*, and set the predicted load current at time *N* to be *y_N_*, and the actual load current to be *y_N_*.

2. User Load Current Zero Crossing Anomaly Detection Results

By comparing the predicted load current *y_N_* with the actual load current *y_N_*, it can be decided whether the user has suspected abnormal electricity consumption. The calculation equation based on sigmoid function [26] is as follows:

(19) $$S_{N}=\left\{ \begin{array}{l} \frac{1}{1+e^{-y_{N}}}\;\;\;\;\;\;\;y_{N}>\;{\bar{y}}_{N}\\ \frac{1}{1+e^{-\mathrm{lg}\frac{y_{N}}{\xi }}}\;\;\;y_{N}\leq {\bar{y}}_{N} \end{array}\right.$$

In Equation (19), *S_N_* is the suspected abnormal score at time *N*. The larger the predicted load current *y_N_*, the greater the *S_N_*, and the greater the possibility of abnormal electricity consumption; the closer the predicted load current *y_N_* is to zero, the smaller the *S_N_*, the less likely the abnormal electricity consumption of its users happens. ξ refers to the opening condition parameter of load current zero crossing anomaly detection, i.e., 0.12 current value of the lowest load used by the user. This research sets the threshold for deciding abnormality to 0.8. When the *S_N_* exceeds 0.8, it will be concluded that the user currently has abnormal electricity consumption.

### 4.3. Abnormal Detection of User Electricity Behavior Based on Isolated Forest Algorithm

Because the research on the abnormal electricity consumption behavior of empty-nest users needs to be based on their electricity consumption behavior, this paper takes the cosine similarity between the user’s electricity consumption curve and its typical electricity consumption curve as a sample for research. The previous abnormal electricity consumption time of empty-nest users is uncertain, hence it is impossible to extract abnormal labels. The isolated forest is an unsupervised fast anomaly detection algorithm based on Ensemble, which has the advantages of low time complexity and high accuracy. It is suitable for detecting the electricity consumption anomaly of empty-nest users [27,28].

The anomaly detection using the isolation forest algorithm is divided into two stages. The first stage is to construct the isolation forest model, which recursively divides the data set until all samples are separated and isolated. The segmentation path of abnormal samples in the isolated forest is short, and can be divided into sub-nodes through a few segmentations; while the segmentation path of normal samples is long, it needs to be divided many times before it can be divided into sub-nodes. Therefore, the algorithm judges whether it is an abnormal object by analyzing the segmentation path length of the sample object.

The second stage is to conduct anomaly detection on the new electricity data of users. It is assumed that the isolated forest model is composed of *T* isolated trees. For the new user data *x*, the segmentation path length *h*(*x*) of data *x* under each isolated tree is calculated. Subsequently, the average segmentation path length *E*(*h*(*x*)) under T isolated trees in the isolated forest is calculated. The smaller the segmentation path length *E*(*h*(*x*)) of the sample in the isolated tree is, the higher the degree of anomaly is.

## 5. Case Analysis

### 5.1. Data Source and Preprocessing

The social information of some electric power users in a certain region of Zhejiang Province is investigated by a questionnaire. The social information includes user family structure, number of people, age, household appliances, and payment methods. The labels of some accurate empty-nesters are obtained by the questionnaire. A total number of 6000 questionnaires was collected after eliminating the questionnaires that did not meet the logical requirements.

According to the household number in the questionnaire, the annual daily power consumption data of users were extracted. It was discovered that the power consumption data that did not meet the requirements were incomplete and duplicated. In this paper, the users with incomplete power data greater than 30% of the annual power data are eliminated. Meanwhile, the users with incomplete power data of less than 30% of the annual power data are compensated by the adjacent missing value linear interpolation method, and the time arrangement method is adopted to remove the duplicate data. After data preprocessing, there are 5254 valid users, 678 empty-nest users, and 4576 non-empty-nest users. The labeled data is divided into a training set and a test set, and the validation set is the data of 2000 unlabeled users who did not participate in the questionnaire, as shown in Table 1.

### 5.2. Empty-Nest Recognition Result Based on Weighted Random Forest Algorithm

The user electricity data is constructed according to the Equations (1)–(3). The training set data is *D*_3520×36_, and the test set data is *D*_1734×36_. To prove the feasibility of the electricity characteristic index, the feature learning method based on principal component analysis (PCA) is introduced as a comparison. Moreover, the feature dimension extracted by PCA is set to be the same as the dimension of the electricity characteristic index database. The cumulative contribution rate of feature information extracted by PCA is 85.96%.

**Figure 5:**
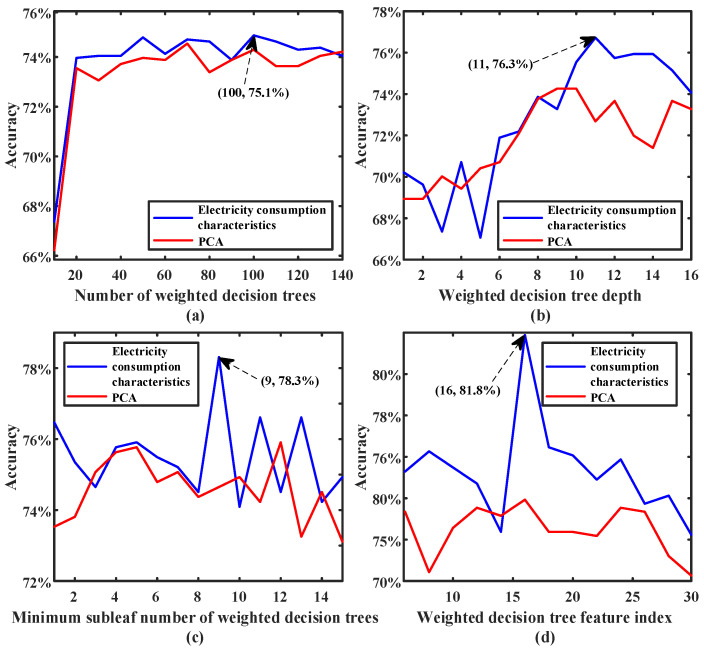
The performance of different features of the weighted random forest under different parameters.

For the optimal model parameters of the weighted random forest, the training set data are trained by 10-fold cross validation, and the average accuracy of empty-nest recognition under cross validation is used as the evaluation index. The performance and model fitting curves of different characteristics of the weighted random forest under different model parameters are shown in Figure 5.

**Figure 6:**
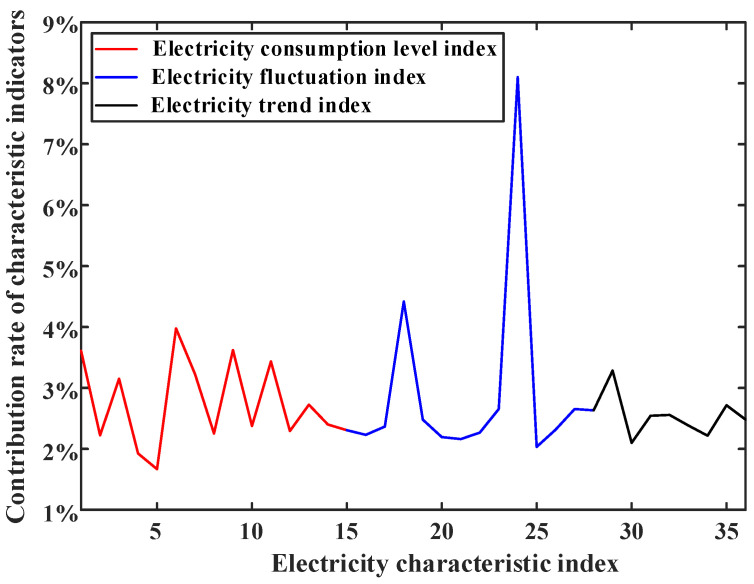
Contribution rate of different electricity characteristics to weighted random forest classifier performance.

It can be seen from Figure 5 that the power consumption characteristic index constructed in this paper is better than the characteristic index based on PCA machine learning. The contribution rate of different power consumption characteristics to the performance of the weighted random forest classifier is shown in Figure 6.

It can be concluded from Figure 6 that the contribution rates of different feature indexes are relatively similar. Each feature has a contribution to the classifier, and the correlation between features is low. There is no situation where the contribution rate of certain types of features is high and the rest of the features are useless features, which shows the feasibility of selecting electricity features in this paper.

**Figure 7:**
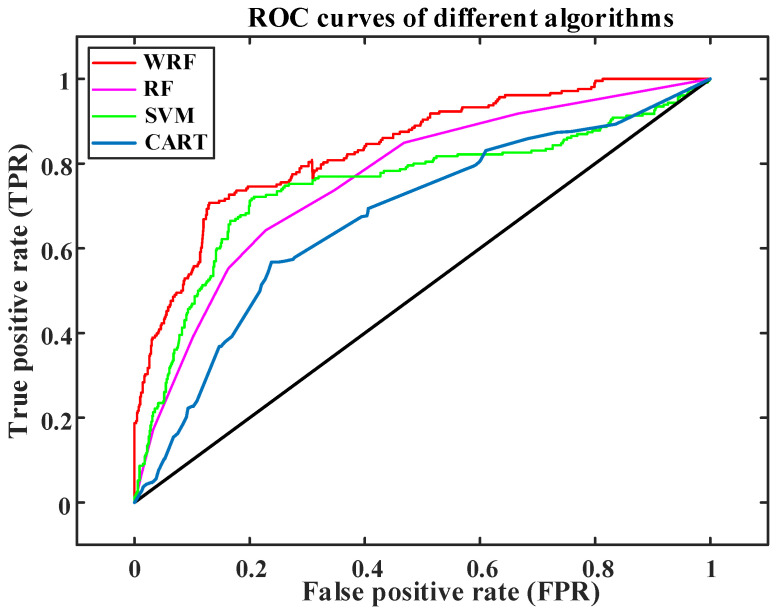
ROC curve of empty-nest user recognition based on different algorithms.

In this paper, a support vector machine, decision tree, random forest, and weighted random forest algorithm are selected for comparison. The comparison of ROC classifier performance evaluation indexes of classification results of different algorithms is shown in Figure 7.

In Figure 7, the ROC curve of WRF is located above other algorithms. It is analyzed that the processing performance of WRF for unbalanced data is better than that of CART, SVM, and RF algorithms, and it has a better recognition ability for empty-nest users. The WRF algorithm is used to establish an empty-nest user recognition model, and the model is deployed on the user acquisition system of a power company in Zhejiang Province.

Using the empty-nest user accurate identification system to identify 2000 unknown users in a certain area of Zhejiang Province, 140 suspected empty-nest elders were identified. According to the field research by the relevant departments of the electric power company, 104 empty-nest users were accurately identified, 36 households were misidentified, and the accuracy rate was 74.2%. According to the principle that the proportion of empty-nest users should be 10%, the empty-nest recall rate is 52%.

There are two main reasons for the inaccurate identification of 36 households in the results. One is that the electricity consumption rules of non-empty-nest users and empty-nest users are basically the same, which leads to the prediction of the user as empty-nest users. Second, since the feature extraction of electricity is not comprehensive or only from the perspective of electricity, it cannot completely construct the empty-nest user feature library, resulting in some non-empty-nest users being predicted as empty-nest users.

### 5.3. Analysis of Electricity Consumption Behavior of Empty-Nest Users Based on Adaptive Cosine K-Means Clustering Evaluation Index

For the accurately identified empty-nest users, the annual daily load current data of the empty-nest user are extracted as the basic data for establishing the typical electricity behavior characteristic curve of the empty-nest elderly. It is assumed that the load current data of the empty-nest user is *D*_365×96_, 365 as the electricity days, and 96 is the number of load data collected every day.

**Figure 8:**
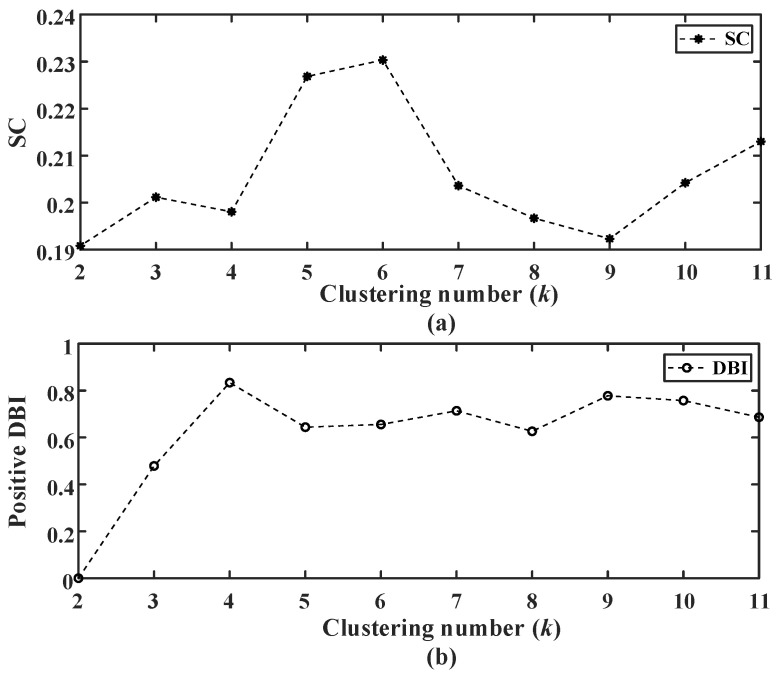
(**a**) SC and (**b**) positive DBI indexes under different clustering number *k*. According to the Equation (17), the positive ideal optimal solution *Z*^+^ is (0.3543, 0.4136), and the negative ideal optimal solution *Z*^−^ is (0.2928, 0). The comprehensive evaluation score calculated by the Equation (18) is shown in Figure 9.

The empty-nest user load after abnormal data processing is clustered by cosine K-means. Since the typical electricity behavior characteristic curve of users is extracted in this paper, it is not typical when the number of days of the extracted curve is less than 10. Therefore, the search threshold *α* of the initial cluster *k* value is set to 10, and the range of cluster *k* value is 2~11. According to Equations (13)–(16), the clustering average contour coefficient SC and DBI indexes under different *k* values are calculated, respectively. The clustering average contour coefficient SC and the DBI indexes after normalization are shown in Figure 8.

**Figure 9:**
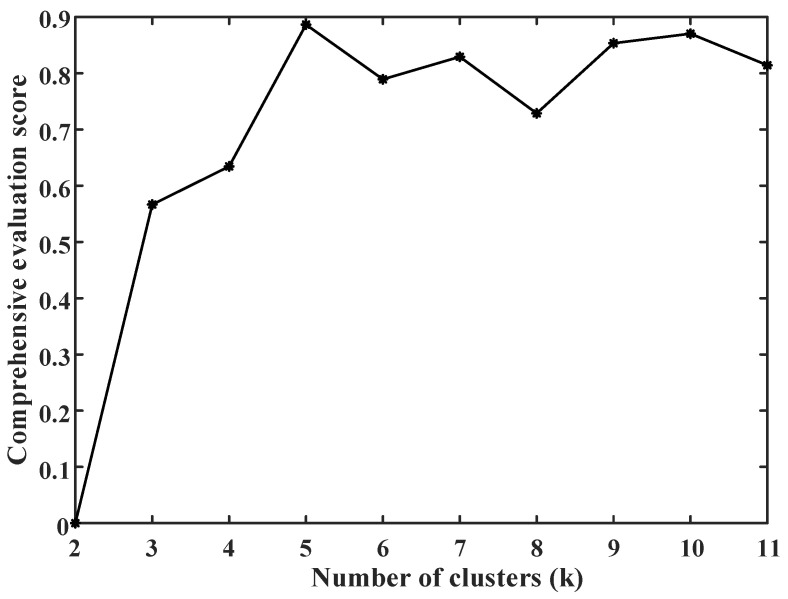
Comprehensive evaluation scores under different clustering number *k*.

**Figure 10:**
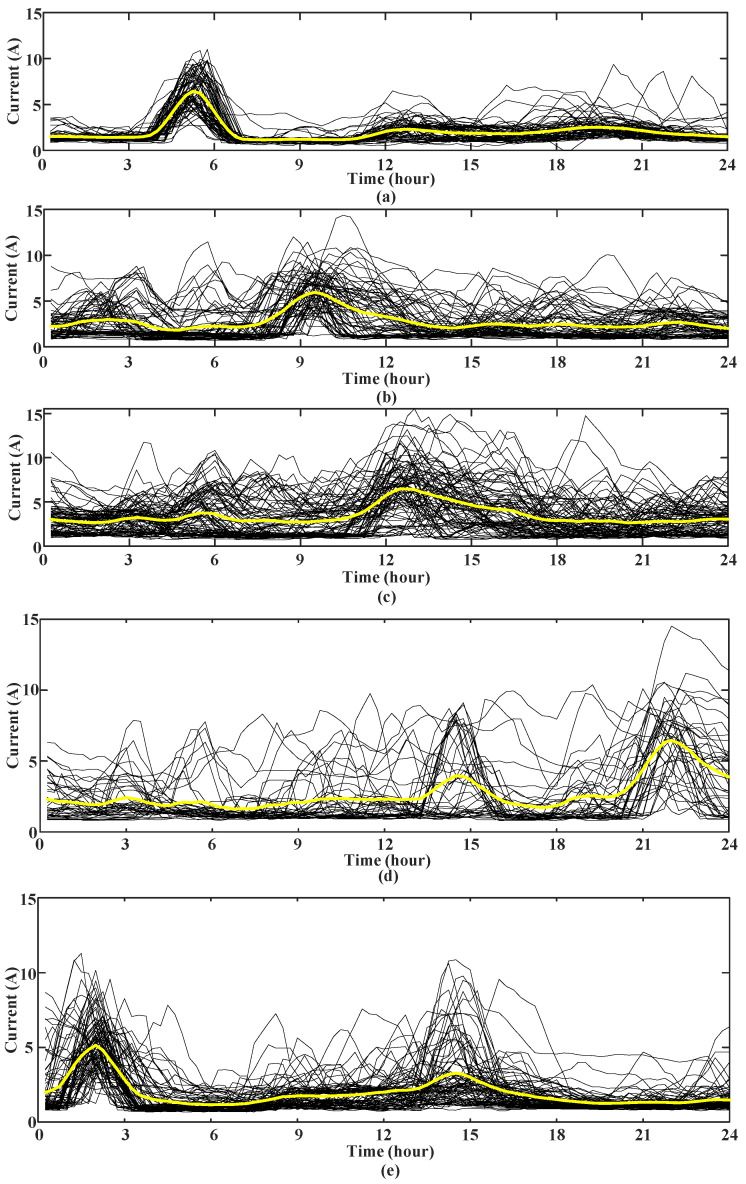
(**a**) The first type, (**b**) the second type, (**c**) the third type, (**d**) the fourth type and (**e**) the fifth type of typical electricity consumption characteristic curves of empty-nest users.

It can be seen from Figure 9 that when the number of clusters *k* = 5, the comprehensive score is the highest. Finally, the clustering result of *k* = 5 is selected, and the typical center characteristic curve of the user is shown in Figure 10.

In Figure 10a, the first typical electricity curve peaked at 5–6 am, which may be caused by the habits of empty-nesters including getting up early to cook. In Figure 10b–d, the second, third and fourth types of typical electricity curves appear electricity peaks in the morning, afternoon, and evening, respectively, which may be caused by the empty-nesters’ rest after meals. In Figure 10e, the fifth type of typical electricity characteristic curve has peak power consumption in the morning and afternoon. It is observed that most of the curve time is in summer and winter, which may be due to the use of high-power electrical equipment such as air conditioning in summer and winter evenings. Through the analysis of five typical electricity consumption curves of empty-nesters, the electricity consumption behavior characteristics reflected by them are consistent with the living habits of empty-nesters in sociology.

In this paper, the K-means algorithm based on the cosine index (C-KM), the K-means algorithm based on the Euclidean metric (EM-KM), the self-organizing maps (SOM) algorithm, and the fuzzy C-means (FCM) algorithm are used to compare and analyze the clustering effect. The number of clustering types is set to 5, and the two clustering results of SSE and MDC are evaluation indexes. MDC represents the average value of the distance between the load curves of different cluster centers. The larger the MDC value, the better the clustering effect. The calculation equation [29] is as follows:

(20) $$\mathrm{MDC}=mean\left(Dist\left(C_{i},C_{j}\right)\right)\;\;\;\;i\neq j$$

*C_i_* and *C_j_* are typical load curves obtained after clustering. The load curves are clustered and analyzed based on the above clustering algorithms, and the comparison results are shown in Table 2.

As can be seen from Table 2, the speed of K-means algorithm is better than SOM algorithm and FCM algorithm. In the study of the electricity consumption behavior of large-scale empty-nest users, the large user base leads to a large scale of power load data. At this time, the advantage of fast K-means algorithm is particularly important. As seen from the SSE and MDC values, the performance of the four clustering algorithms from high to low is C-KM, EM-KM, SOM and FCM.

### 5.4. Research Results of Abnormal Electricity Consumption Monitoring for Empty-Nest Users

#### 5.4.1. Forecast Model of Load Current Based on ARIMA Algorithm

**Figure 11:**
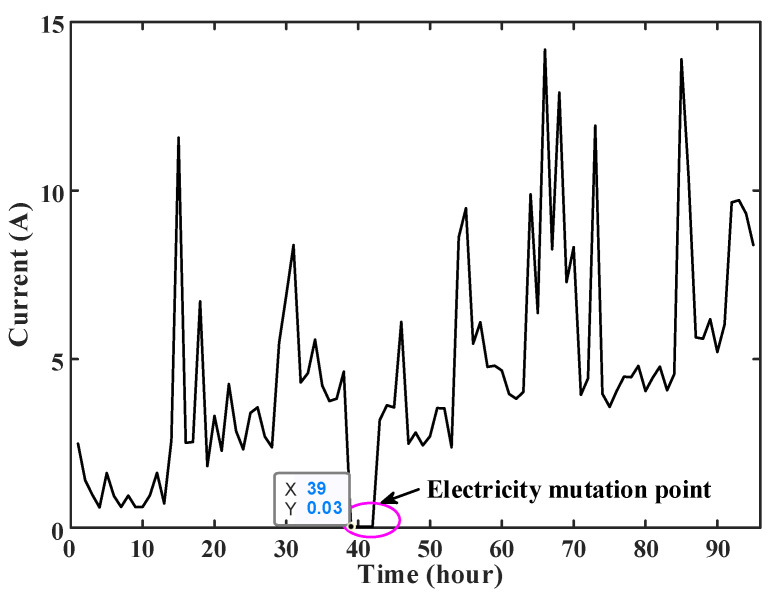
Abnormal electricity consumption diagram for users suspected of sudden change in load current.

The abnormal power consumption at the zero-crossing point of load current was detected for an empty-nest user in a certain area of Zhejiang Province. It was discovered that the abnormal power consumption of the empty-nest user occurred one day in August 2021, and the load current changed to 0.03 A at the 39th point, as shown in Figure 11.

At this moment, the current 0.03 A is lower than the opening condition 0.12 A of the current zero-crossing anomaly detection. The load data of the user in the previous week are extracted to construct the ARIMA (*p*, *d*, *q*) model to predict the load current at this time. After the steps of smoothing treatment and model order, the ARIMA (9, 1, 8) model is constructed to predict the load current at the 39th point at the anomaly time, and the predicted current value *y*_39_ should be 4.621 A. According to Equation (19), the suspected anomaly score *S_N_* is 0.990, which determines that the suspected abnormal electricity of this user has happened. After the telephone survey of the user conducted by the power company, it is discovered that the user has abnormal electricity.

The same method was applied to detect all empty-nest users in this area. A total of 37 households were detected to have abnormal power consumption at zero-crossing point of load current from August to October 2021. There are 32 accurate detection households, and the accuracy rate was 86%. One of the five households with identification errors was caused by the power outage of the family, and the other four households were due to model identification errors.

#### 5.4.2. Monitoring Results of Empty-Nest Abnormal Electricity Consumption Behavior Based on Isolated Forest Algorithm

The daily load current data *D* = {*d*_1_, *d*_2_, … *d*_365_} ∈ *R^s^* of an empty-nest user in Zhejiang in 2020 were extracted. The user load current data collection frequency was once every 15 min, and a total of 96 points were collected every day. The adaptive cosine K-means based on the fusion clustering evaluation index is used to analyze the electricity consumption behavior of empty-nest users. A total of five types of typical electricity load curves are obtained. The cosine similarity between the daily electricity curve in dataset *D* and the *k* type of typical curve is calculated to construct the anomaly detection model dataset *I*_365×6_.

**Figure 12:**
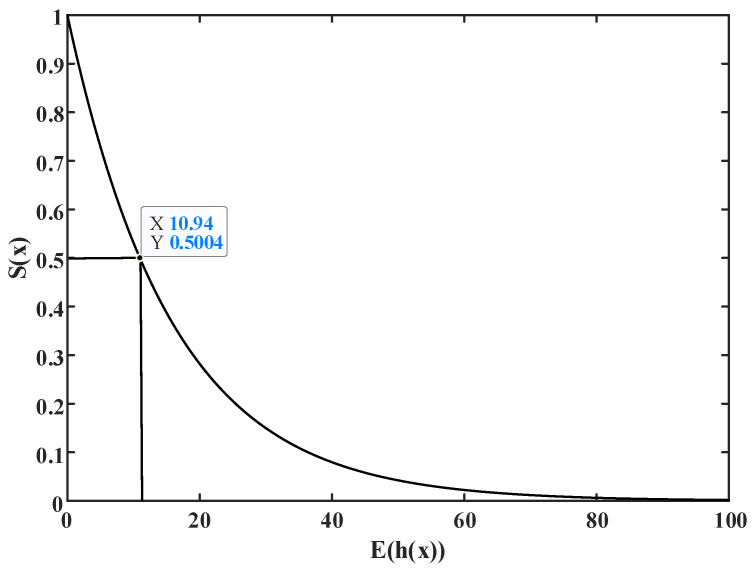
Distribution of abnormal scores of empty-nest users.

The outlier detection model of an isolated forest is constructed based on data set *I*. The parameters of the isolated forest are as follows: 128-day data are randomly selected by the bootstrap sampling method. Build *T* = 100 isolated tree models. The average segmentation path length *c*(*n*) of the isolated forest model is calculated as 10.9542. The user’s daily real-time electricity consumption data after 1 January 2021 are used to calculate the anomaly score *s* by the isolated forest anomaly detection model. When *s* is greater than 0.8, it is determined that the user’s behavior is abnormal. The distribution of the anomaly score *s* is shown in Figure 12.

**Figure 13:**
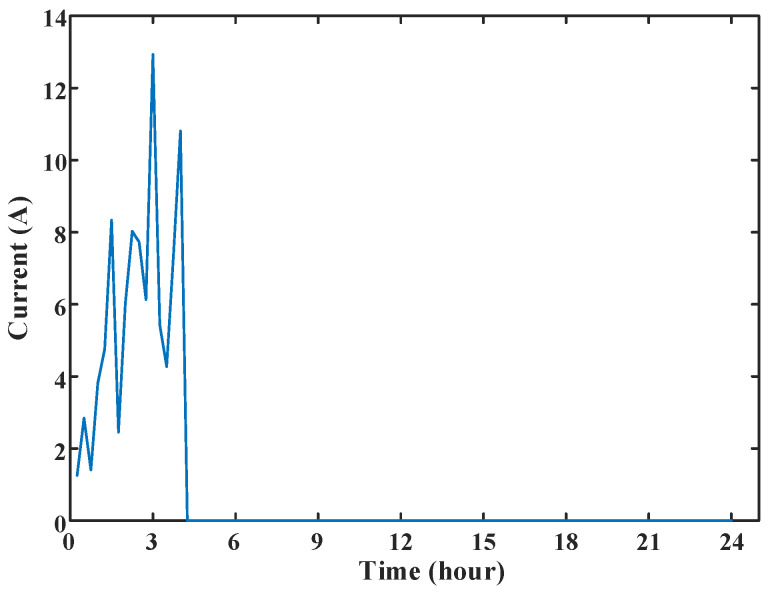
User’s actual load current curve on the day.

On 3 April 2021, the user’s abnormal score *s* on the day was equal to 0.912, which was greater than 0.8. It could be concluded that the user’s electricity behavior was abnormal on that day, and it was reported to the relevant power departments. After verification by the relevant departments, it was found that the empty-nest old user’s home was cut off at about 4:00 a.m. due to a power switch problem, while the empty-nest older was unable to repair by themself due to their age. The power maintenance personnel helped them repair the power switch in time and reworked the circuit, eliminating the potential safety hazard of electricity. The electricity consumption curve of the user is shown in Figure 13.

It can be summarized from the example analysis that the detection method for abnormal electricity consumption of empty-nesters proposed in this paper is feasible. The power company investigates the electricity consumption habits of empty-nesters through power data mining, realizes the monitoring of abnormal power consumption of empty-nesters, and provides personalized and differentiated services for empty-nesters in power use. Moreover, it can establish contact with the children of the older empty-nesters. For empty-nest elderly users who have found abnormal electricity use, they should notify their children by phone or SMS in time to help them avoid electricity safety risks.

## 6. Conclusions

This paper constructs an empty-nest user identification model based on weighted random forest and uses the data in the electricity information collection system to identify empty-nest users, which can save manpower and material resources compared with field surveys and other methods. For the accurately identified empty-nest users, an analysis model of empty-nest users’ electricity consumption behavior based on clustering index fusion adaptive cosine K-means is proposed, and the characteristics of empty-nest users’ electricity consumption behavior are mined and analyzed from the perspective of social behavior. We explained and improved the electricity consumption portrait of the empty-nest user group. At the same time, based on the past electricity consumption data of empty-nest users, ARIMA and the isolated forest algorithm are used to establish an abnormal electricity consumption detection model, and the daily life and electricity consumption behavior of empty-nest elderly people are detected from the perspective of electricity consumption. The results show that the proposed method can effectively help them avoid electricity risks. It is not possible to fully construct the feature database of empty-nest users only from the perspective of electricity consumption. Therefore, in the follow-up research, the marketing data of the power sector can be added, such as the payment channel of the user’s electricity bill, arrears records, the registration information of electric meters, and information on the head of household. It is beneficial to strengthen the basis for group classification and improve the accuracy of empty-nest user identification. The abnormal power consumption detection model for the empty-nester elderly is currently unable to avoid the impact of random factors such as weather, holidays, and family member regression. In the future, the deviation caused by these accidental factors can be reduced by giving tolerance factors or reducing the weight. It can increase the generalization ability and robustness of the anomaly detection model, thus improving the recognition ability of sudden changes in power consumption behavior caused by power consumption risks.

## Figures and Tables

**Table 1:** User data of training set and test set.

	Non-Empty-Nest	Empty-Nest	Sum
Training set	3055	465	3520
Test set	1521	213	1734
Sum	4576	678	5254
Validation set	/	/	2000

**Table 2:** Performance comparison of four algorithms.

Algorithm	Time (s)	SSE	MDC
C-KM	3.4281	31.6591	13.9513
EM-KM	3.4478	165.2100	9.6585
SOM	4.5721	161.3024	2.2093
FCM	12.8700	780.4917	12.9373

## Data Availability

The data presented in this study are available on request from the corresponding author. The data are not publicly available due to confidentiality.
